# Elevation of Oxidized Lipoprotein of Low Density in Users of Combined Oral Contraceptives

**DOI:** 10.5935/abc.20180194

**Published:** 2018-12

**Authors:** Alan Carlos Nery dos Santos, Jefferson Petto, Diego Passos Diogo, Candice Rocha Seixas, Lunara Horn de Souza, Wagner Santos Araújo, Ana Marice Teixeira Ladeia

**Affiliations:** 1 Escola Bahiana de Medicina e Saúde Pública, Salvador, BA - Brazil; 2 Universidade Salvador (UNIFACS), Feira de Santana, BA - Brazil; 3 Faculdade Adventista da Bahia (FADBA), Cachoeira, BA - Brazil; 4 Faculdade Social da Bahia (FSBA), Salvador, BA - Brazil

**Keywords:** Cardiovascular Diseases/complications, Contraceptives, Oral, Combined, Lipid Metabolism Disorders, Oxidative Stress, Atherosclerosis, C-Reactive Protein

## Abstract

**Background:**

The use of combined oral contraceptive (COC) has been related to changes in glycemic, lipid metabolism, increased oxidative stress, and systemic blood pressure, which could suggest a higher oxidation of low-density lipoprotein cholesterol (LDL-cholesterol) in women on use of COC.

**Objective:**

To test the hypothesis that there is a difference in the plasma values of oxidized LDL among women who use and do not use COC, as well as to evaluate the correlation between it and the lipid profile and high-sensitivity C-reactive protein (hs-CRP).

**Methods:**

Forty-two women with ages between 18 and 35 years old, who were eutrophic, irregularly active, with triglycerides < 150 mg/dL, blood glucose < 100 mg/dL, and who used or did not use COC were selected. These women were allocated in the COC group, formed by 21 women on COC use for at least 1 year; and a control group (CG), consisting of 21 women who had not used any type of hormonal contraceptive for at least 1 year. A significance level of 5% was adopted for statistical analyses.

**Results:**

It was observed that GCOC showed higher values of oxidized LDL than the CG, respectively 384 mU/mL versus 283 mU/mL (p < 0.01). A positive correlation between oxidized LDL and LDL-cholesterol (r = 0.3, p < 0.05), with total cholesterol (r = 0.47, p < 0.01) and with triglycerides (r = 0.32, p < 0.03) was observed, and there was no correlation with the hs-CRP. In the categorized analysis of oxidized LDL, 71.4% of GCOC women, and 28.6% of the CG remained above the established cutoff point.

**Conclusion:**

Women who use COC have higher plasma levels of oxidized LDL, and there is a positive correlation between oxidized LDL and other lipid variables.

## Introduction

Studies have shown that women of reproductive age who use combined oral contraceptives (COCs) present changes in glycemic,^1^ lipid metabolism,^2^ oxidative stress,^3^ and chronic subclinical inflammation.^4,5^ Also, an increase in the atherogenic subfractions of low-density lipoprotein (LDL-cholesterol)^6^ and elevated systemic blood pressure (SBP)^7^ were identified. Together, these alterations are associated with LDL-cholesterol oxidation, which has been strongly related to a more atherogenic lipid profile.^8^

Once oxidized, LDL-cholesterol presents several actions in vascular physiology, among them, it inhibits the expression of the endothelial nitric oxide synthetase enzyme mRNA, resulting in a decrease in the production of nitric oxide and favoring the atherosclerotic process.^9^ Furthermore, it also impairs cell proliferation, cell motility and endothelial stem cells action, which are key mechanisms in the endothelialization of damaged areas in the atherosclerotic process.^10,11^ It has also been suggested that higher oxidized LDL values, even within the limits of normal, are associated with an increased risk of future cardiovascular events and metabolic syndrome.^1,12-14^

In addition, in Brazil, 33.8% of women aged 18-49 years used oral contraceptives, and of these, more than 13% (95% CI, 10.9-15.7%) had risk factors, such as smoking, systemic arterial hypertension, dyslipidemias and obesity.^15^ These factors, associated with the use of COCs, can significantly increase the risk of atherothrombotic events, even in women of reproductive age.^16,17^

However, to our knowledge, there are still no studies that have investigated the oxidation of LDL-cholesterol in young women using COC, without other factors that justify their oxidation. Thus, the hypothesis that there is a difference in the plasma values of oxidized LDL among women who use and do not use COC was tested, and the correlation between oxidized LDL and the fasting lipid profile variables and C-reactive protein were evaluated.

## Methods

### Sample

The research is characterized as a cross-sectional analytical study, which has as a predictor variable the use of COC, and as an outcome variable, the oxidized LDL.

The study population consisted of 42 self-reported healthy, eutrophic, irregularly active women aged 19 to 30 years, nulliparous, with fasting values of triglycerides < 150 mg/dL, blood glucose < 100 mg/dL, and who used COC or not. All participants were students of a private college located in the city of Salvador, BA - Brazil.

The sample was divided into two groups: COC group (GCOC) consisting of 21 women using COC of low dose of ethinylestradiol (15 to 30 mcg) for at least 1 year; and control group (CG), consisting of 21 women who had not used any type of hormonal contraceptive for at least 1 year.

To determine if participants were irregularly active, the International Physical Activity Questionnaire (long version), developed by the World Health Organization and the US Centers for Disease Control and Prevention was used.^18^

Women who reported familial dyslipidemia, hypo- or hyperthyroidism, history of alcoholism or smoking, polycystic ovarian syndrome, hypo- or hyperlipidic diet, use of dietary or anabolic supplements, hypolipidemic agents, corticosteroids, diuretics or beta blockers were excluded. Those who presented, on the physical evaluation, values of SBP ≥ 140/90 mmHg, abdominal circumference ≥ 80 cm or, in the laboratory examination, alteration of pyruvic (TGP) or oxidative (TGO) glutamic transaminase, or creatinine were also excluded. TGP and TGO were evaluated to identify pancreatic and hepatic diseases, and creatinine, to identify the presence of renal dysfunction.

All the participants answered the semi-structured questionnaire, elaborated by the authors of the research and underwent physical examination. The latter consisted of resting blood pressure (BP), total body mass, height and waist circumference.

Body mass index (BMI) was calculated with mass and height measurements, according to the Quetelet equation: mass (kg)/height^2^ (cm). The BMI cutoff points adopted were those recommended by the IV Brazilian Guidelines on Dyslipidemias and Prevention of Atherosclerosis of the Department of Atherosclerosis of the Brazilian Society of Cardiology (SBC),^19^ that is, low weight (BMI < 18.5); eutrophy (BMI 18.5-24.9); overweight (BMI 25-29.9), and obesity (BMI ≥ 30).

The abdominal circumference was obtained with a Starrett^®^metric and inelastic tape, with a measurement definition of 0.1 cm. It was measured at the lowest curvature located between the last rib and the iliac crest without compressing the tissues.^20^

### Laboratory Data Collection Protocol

To collect the laboratory data, the volunteers were referred to the Laboratory of Clinical Pathology in the city of Salvador, state of Bahia - Brazil, where blood samples were collected. Following antecubital vein puncture, 10 mL of blood were collected for triglycerides (TG), oxidized LDL, high-density lipoprotein (HDL-cholesterol), total cholesterol (TC), blood glucose, pyruvic glutamic and oxidative transaminase. LDL-cholesterol and the very low density cholesterol (VLDL-cholesterol) were calculated by the Friedewald equation:^21^ TC = HDL-cholesterol + LDL-cholesterol + VLDL-cholesterol, with VLDL-cholesterol being equal to TG/5.

The collections were performed with the volunteers fasting for 12 hours. All were instructed not to change their diet during the week of the test, not to perform any physical exertion other than usual, and not to drink alcoholic beverages 24 hours before the laboratory examination. For the CG, the collections were performed between the 5th and 10th day of the menstrual cycle, considering the lower hormonal fluctuations, as recommended by Casazza et al.^22^ Blood samples were collected by a trained professional and in a laboratory environment suitable for this type of procedure.

For determination of oxidized LDL in the serum samples, the ELISA kit was used. In this analysis, the oxidized LDL values considered normal were between 100 and 700 mU/mL. The triglycerides, HDL-cholesterol, total cholesterol and blood glucose values were obtained by the Trinder colorimetric enzyme method.^23^ TGP and TGO were measured by the Reitman-Frankel colorimetric method.^24^

The sample adequacy calculation was performed in the GraphPad StatMate 2.0 for Windows software, which considered a difference between the means of 63 MU/mL and standard deviations of 119.5MU/mL (GCOC) and 43.6 MU/mL (CG), both extracted from a previous pilot study (n = 12). In order to eliminate the bias of the laboratory variation coefficient of oxidized LDL dosage, which was of 3%, a significant difference was considered between the groups, of 20% for alpha and beta of 0.05 (bidirectional) and 0.80, respectively. Thus, 20 women were needed in each group.

### Statistical analysis

Initially, symmetry and kurtosis tests and the Shapiro-Wilk test were applied to check data distribution. The variables values with normal behavior were described in mean and standard deviation and the values of nonparametric variables in median and interquartile range. Categorical variables were presented as absolute and relative frequencies.

For the intergroup comparison of the parametric variables, we used the unpaired bidirectional Student t test, and for the non-parametric variables, the Mann-Whitney test. The correlation between the oxidized LDL values and all variables of the lipid profile - triglycerides, total cholesterol, HDL-cholesterol and LDL-cholesterol, and CRP was also verified. In the correlation analysis, the Spearman correlation coefficient was used.

In addition to the inter-group comparisons of oxidized LDL, the sample was categorized based on the median of oxidized LDL in women with LDL-oxidized values above and below the median. After the categorization, Fisher's exact test was used. All analyzes were performed in the BioStat 5.0 statistical package, adopting a significance level of 5%.

### Ethical aspects

Throughout the study the guidelines on human research in the Declaration of Helsinki and Resolution 466/12 of the National Health Council were followed. This study was submitted and approved by the Research Ethics Committee of Faculdade de Tecnologia e Ciência de Salvador - BA with number 3.390/2011.

All participants received detailed information about the study objectives, risks and benefits involved in the procedures and signed the informed consent form. Two copies were filled, one being kept with the participants, and the other with the researchers.

## Results

Table 1 presents the clinical and anthropometric characteristics of the sample. Homogeneity between the groups is observed, and the difference between the values of the SBP (p < 0.02) and the CRP (p < 0.01) are highlighted, which are higher in the GCOC.

**Table 1:** Clinical and anthropometric characteristics of women using and not using combined oral contraceptives (n = 42)

Variables	GCOC (n = 21)	CG (n = 21)	p value
Age (years)	23 ± 3.1	23 ± 3.4	0.98*
Body mass index (kg/m^2^)	20 ± 2.1	19 ± 2.8	0.07*
Waist circumference (cm)	73 ± 7.8	70 ± 5.9	0.32*
Systolic blood pressure (mmHg)	118 ± 8.8	111 ± 9.7	0.02*
Diastolic blood pressure (mmHg)	77 (74 – 80)	70 (70 – 80)	0.18**
C-reactive protein (mg/L)	2.7 (1.8 – 6.4)	0.9 (0.5 – 1.1)	< 0.01 **
Blood glucose (mg/dL)	82 ± 6.9	83 ± 5.7	0.57*
Pyruvic glutamic Transaminase (U/L)	15 ± 4.2	14 ± 3.4	0.16*
Time of use of COC (years)	3.7 ± 2.3	–	–

GCOC: combined oral contraceptive group; CG: control group; COC: combined oral contraceptive;

* Bidirectional Student’s t test for independent samples;

** Bidirectional Mann-Whitney test.

When comparing the lipid fasting variables, and the TG/HDL-cholesterol ratio (Table 2), it is observed that the GCOC presents values of plasma triglycerides (p < 0,01), total cholesterol (p < 0,01), HDL-cholesterol (p < 0,04), VLDL-cholesterol (p < 0,01) and TG/HDL-cholesterol ratio (p < 0,01) higher than the GC.

**Table 2:** Comparison of fasting lipids (mg/dL) among the groups studied

Variables	GCOC (n = 21)	CG (n = 21)	p value
Triglycerides	95 (73 – 112)	49 (40 – 64)	< 0.01 **
Total cholesterol	210 ± 38.6	183 ± 29.7	0.01*
HDL-c	58 ± 19.3	48 ± 11.5	0.04*
LDL-c	134 ± 35.1	126 ± 27.7	0.42*
VLDL-c	19 (15 – 22)	10 (8 – 13)	< 0.01 **
TG/HDL-c ratio	1.7 ± 0.5	1.1 ± 0.5	< 0.01*

GCOC: combined oral contraceptive group; CG: control group; HDL-cholesterol: high-density lipoprotein cholesterol; LDL-cholesterol: low-density lipoprotein cholesterol; VLDL-cholesterol: very low-density lipoprotein cholesterol;

* Two-way t-test for independent samples;

** Bidirectional Mann-Whitney test.

**Figure 1:**
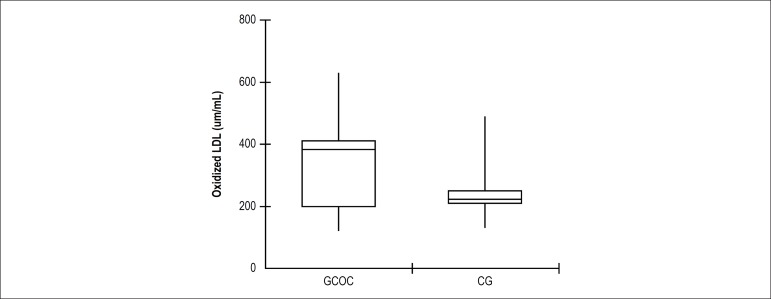
The boxplot shows a higher concentration of oxidized LDL in women using combined oral contraceptives compared to those who did not use this group of drugs (p < 0.01). In addition, it is noted that in the GCOC the concentration of this oxidized lipoprotein is in the first quartile, while the CG is in the third quartile. The comparison of the median between groups was compared by bidirectional Mann-Whitney test.

As shown in Figure 1, GCOC women had higher oxidized LDL plasma levels (mU/mL) than the CG, 384 (198-410) versus 283 (208-250) (p < 0.01).

In Table 3, the analyses of correlation between oxidized LDL and the variables of the fasting lipid profile, as well as between oxidized LDL and the PCR are presented. Moderate and positive linear correlation was observed between oxidized LDL, and LDL-cholesterol, triglycerides and total cholesterol.

**Table 3:** Correlation analysis between LDL-oxidized (mU/mL) and fasting lipid profile variables (mg/dL) and CRP (mg/dL)

Crossings	Correlation coefficient (rs)	p value*
Oxidized LDL and TG	0.32	0.03
Oxidized LDL and CT	0.47	< 0.01
Oxidized LDL and LDL-cholesterol	0.29	0.05
Oxidized LDL and HDL-cholesterol	0.26	0.08
Oxidized LDL and PCR	0.20	0.19

Oxidized LDL: oxidized low-density lipoprotein; TG: triglycerides; TC: total cholesterol; HDL-cholesterol: high-density lipoprotein cholesterol; LDL-cholesterol: low-density lipoprotein cholesterol; CRP: C-reactive protein;

* Spearman's correlation test.

In Table 4, we can observe the intergroup analysis of oxidized LDL when categorized based on the value of the median. It can be seen that 71.4% of the women in the GCOC had higher plasma oxidized LDL values than the established cut-off when compared to the CG, which was 28.6% (p < 0.01).

**Table 4:** Categorical analysis based on the median of oxidized LDL

		COC		p value*
		No n (%)	Yes n (%)	
Oxidized LDL	< 247	15 (71.4%)	6 (28.6%)	< 0.01
	> 247	6 (28.6%)	15 (71.4%)	

Oxidized LDL: oxidized low-density lipoprotein; COC: combined oral contraceptive.

* Fisher’s exact test.

## Discussion

In response to the objectives of this study, we identified that women who use COC have higher oxidized LDL values, with a moderate and positive correlation of oxidized LDL with LDL-cholesterol, total cholesterol and triglycerides. In addition, 71.4% of the women who used COC presented oxidized LDL values above the cutoff point when compared to the control group (28.6%). Thus, although it is not possible to establish a perfect cause-effect relationship due to the method used, to the non-stratification of COC types, and to the effects of regionality, the results presented here are reinforced by the characteristics and homogeneity of the sample, which does not present the classic factors that could be known to induce the increase of oxidized LDL. In this context, although there is no clearly defined mechanism, some hypotheses may explain the elevation of oxidized LDL in women who use COC.

It should be noted that, in recent years, scientific evidence has increasingly made the role of oxidized LDL in the pathophysiology of atherosclerosis clearer.^25^ However, there is still no clearly defined mechanism, but several hypotheses that help explain the oxidation of LDL-cholesterol in different populations.^8,25^ One of these hypotheses demonstrates that the bioavailability of LDL-cholesterol in association with oxidative stress appears to be the main determinant for the formation of oxidized LDL.^8^

Thus, although we did not observe a difference in the fasting LDL-cholesterol levels among the groups studied, we suggest that the GCOC has a higher concentration of the more atherogenic LDL-cholesterol subfraction. This particle is small and dense, and has lower concentrations of antioxidants. Taken together, these factors make it more prone to oxidative damage.^26^ In this study, the hypothesis in question is based on the TG/HDL-cholesterol ratio result, which we found to be significantly higher in GCOC. In addition, it has been suggested that the TG/HDL-cholesterol ratio may reflect the size of LDL-cholesterol particles, with values > 1 being indicative of small and dense particles.^26^ Consistent with our study, Graaf et al.^6^ showed that women who use COC have higher concentrations of atherogenic LDL-cholesterol subfraction, which may suggest a more atherogenic lipid profile in this population.

In contrast to our findings, although in a population of 40-48 years of age, different oral contraceptive formulations, and factors such as smoking, intestinal disease and physical activity, the ELAN study^3^ did not identify any significant changes in plasma oxidized LDL of women who use and do not use oral contraceptives. However, it was noted that in women using this group of drugs, the lipid oxidation, marked by the highest concentration of peroxides (-OOH), was 1.7 times higher. According to the authors, this result could be explained by the higher oxidative stress induced by ethinylistradiol present in the formulations of COC.^3^

In line with this observation, we can suggest, as well as other studies, that women on COC have higher oxidative stress.^3^ This hypothesis can be supported by the significant increase in oxidized LDL in GCOC, because according to the literature this oxidized lipoprotein is a variable of oxidative stress.

According to literature data, the estrogenic and androgenic properties of COCs have an influence on oxidative stress, because these hormones have several actions on the vascular endothelium, increasing the bioavailability of nitric oxide, a fact that does not seem to protect, but rather attacks the endothelium, due to increased oxidative stress.^27^

Another fact that calls attention is that oxidized LDL has a correlation with other lipid variables. In fact, our results, as well as other studies, indicate that oxidized LDL has a moderate positive correlation with total cholesterol, triglycerides and LDL.^8,12^ This relationship may be partially justified by findings indicating that an increase of 1mg/dL in serum levels of total cholesterol or LDL-cholesterol, as well as an increase of one unit in the total cholesterol/HDL-cholesterol ratio, can predict increases of 0.22, 12.21 and 15.78 U/L at oxidized LDL levels.^28^ According to the literature, triglycerides can predict, regardless of variables such as LDL-cholesterol, elevated oxidized LDL values.^27^

Consistent with the literature, our study demonstrated a significant increase in serum TG, HDL-cholesterol, CRP, and systolic blood pressure values in GCOC, whereas no difference was detected in LDL-cholesterol values.^29-31^ However, caution should be taken when analyzing the LDL-cholesterol and HDL-cholesterol results, because the TG/HDL-cholesterol ratio is significantly higher in this group of women, indicating a higher atherogenic potential related to LDL cholesterol. Regarding HDL cholesterol, although in our sample its values are significantly high, it is not yet known what the effects of COC on its subfractions are, since atherogenic particles of HDL-cholesterol are present.^32^

It is also interesting to note that the use of COC has been suggested as an independent factor for plasma CRP elevation in women of reproductive age. This increase appears to be associated with changes in estrogen β receptor function and levels, increased cortisol, increased TNF-α, hypomethylation in the DNA of macrophages, and alterations in hepatic PCR synthesis. It is also worth noting that the current use of COC can independently represent 20 to 32% of the variation of CRP in these women.^33^ In addition, it was also shown that one in three women on COC shows CRP > 3 mg/L, which according to the literature can markedly increase the risk of cardiovascular events.^29^

In addition, as in our results, research has shown a significant elevation of blood pressure in women on use of COC.^7,34,35^ In fact, according to some studies, COC use may be related to mild and moderate arterial hypertension, with increases ranging from 20 to 40 mmHg in SBP and 10 to 20 mmHg in the diastolic pressure. Also, according to the studies, this elevation can be reversed within 3 months after COC descontinuation.^34^ Such elevation of blood pressure may occur due to changes in electrolyte concentrations, oxidative stress, insulin resistance, and increased production of renin and hepatic angiotensinogen in these women.^34,35^

Therefore, in addition to the fact that oxidized LDL emerges as a non-traditional risk factor for future cardiovascular events in postmenopausal women,^14^ and that, in the pathophysiology of atherosclerosis, besides being present in all stages of the atherosclerotic process, it begins to be deposited in the arterial wall of young adults, even before the initial formation of the atheromatous plaque,^36^ it is suggested that women taking COC present a greater future cardiovascular risk than women who do not use this group of drugs.

Oxidation of LDL-cholesterol is closely related to endothelial dysfunction in a positive feedback process. The endothelial dysfunction associated with the arterial vascular inflammatory process are mainly responsible for the oxidation of LDL cholesterol, which in turn causes endothelial cell toxicity and chemotactic attraction of monocytes/macrophages through feedback of endothelial dysfunction. This mechanism is known as the oxidative theory of atherogenesis.^37,38^

The results presented here point to mechanisms that may help elucidate the outcome of a multicenter study that showed that COC use is associated with a 5-fold increased risk of myocardial infarction in Europe and more than 4-fold in non-European countries. It is worth mentioning that this increase is closely linked to COC formulations with estrogen (≥ 50 µg), and the presence of classic risk factors such as smoking, hypertension, dyslipidemia, and obesity.^16,17^ Another interesting study showed that women taking COC with ethinyl estradiol dosages between 30 and 40 µg had a risk of arterial thrombosis between 1.3 and 2.3. At lower dosages (20 µg), the risk was 0.9 and 1.7 times, when compared to women who did not use this group of drugs. These results suggest that even at low dosages, COCs may increase the risk of atherothrombosis, a fact that should be taken into consideration during its prescription, especially in women presenting cardiovascular and metabolic disease risk factors.^17,39^

Finally, the present study has limitations that need to be discussed. One of them is the non-stratification of COC types. Although being of 3rd generation, COC has different formulations in concentrations of estrogen and progestin, a fact that, in addition to being able to cause different effects on the metabolism, limits the generalization of the results as to the type of hormone present in the formulation of contraceptives. In addition, dietary control was not adequately performed, although we did not select volunteers in control or dietary limitation, and the influence of diet on our results cannot be completely excluded. It is also important to point out that the limitations presented do not impair the results of this study. On the contrary, they add data that facilitate the understanding of alterations in the lipid profile of women of reproductive age who use COC.

## Conclusion

In summary, the findings of this study indicate that women who use COC have a significant increase in plasma oxidized LDL values, as well as higher concentrations of small and dense LDL-cholesterol subfractures, identified by the TG/HDL-cholesterol ratio. We also identified a moderate and positive correlation of oxidized LDL with atherogenic variables of the lipid profile, which may suggest a greater vascular aggression and, consequently, a higher cardiovascular risk in this population. Finally, we can also suggest higher oxidative stress, represented indirectly by the higher concentration of oxidized LDL in these women.
